# Assistive Handlebar Based on Tactile Sensors: Control Inputs and Human Factors

**DOI:** 10.3390/s18082471

**Published:** 2018-07-30

**Authors:** Andrés Trujillo-León, Wael Bachta, Julián Castellanos-Ramos, Fernando Vidal-Verdú

**Affiliations:** 1 Department of Electronics, Institute of Biomedical Research of Málaga (IBIMA), University of Málaga, 29071 Málaga, Spain; jcramos@uma.es (J.C.-R.); fvidal@uma.es (F.V.-V.); 2 Sorbonne Université, Institut des Systèmes Intelligents et de Robotique UMR 7222 CNRS, ERL INSERM U1150 Agathe, 75005 Paris, France; wael.bachta@sorbonne-universite.fr

**Keywords:** tactile sensors, assistive technology, user interface, wheelchairs, attendant, human factors

## Abstract

Tactile sensors can be used to build human-machine interfaces, for instance in isometric joysticks or handlebars. When used as input sensor device for control, questions arise related to the contact with the human, which involve ergonomic aspects. This paper focuses on the example application of driving a powered wheelchair as attendant. Since other proposals use force and torque sensors as control input variables, this paper explores the relationship between these variables and others obtained from the tactile sensor. For this purpose, a handlebar is instrumented with tactile sensors and a 6-axis force torque sensor. Several experiments are carried out with this handlebar mounted on a wheelchair and also fixed to a table. It is seen that it is possible to obtain variables well correlated with those provided by force and torque sensors. However, it is necessary to contemplate the influence of issues such as the gripping force of the human hand on the sensor or the different kinds of grasps due to different physical constitutions of humans and to the inherent random nature of the grasp. Moreover, it is seen that a first step is necessary where the contact with the hands has to stabilize, and its characteristics and settle time are obtained.

## 1. Introduction

There is an increasing concern in developed societies regarding population aging. Moreover, sensitivity to inclusion of people with disabilities also pushes the research and investment in assistive technologies. This paper focuses on the attendant driving interface for powered wheelchairs as example application, though the conclusions apply to other systems such as trolleys [1], or smart walkers [2]. The attendant is required when the user is not able to drive the chair on his or her own, for instance because of cognitive impairment. The commercial standard solution is the use of a joystick at the rear of the chair. However, this device is not intuitive to use [3], and it is better to implement interfaces closer to those that people use daily to drive trolleys or similar systems, such as handlebars. Several robotic wheelchairs or trolleys have been developed with force and torque sensors embedded in the handlebar to register the force and torque exerted by the driver on it as control inputs [4,5,6]. The authors of this paper proposed the use of tactile sensors instead of force sensors for that purpose. This technology was presented in [3,7], and tests to show its feasibility were reported in those works. The aim of this paper is to cover a few key aspects of the tactile handlebar as steering device that the authors think can be certainly useful for researchers and engineers who cope with the development of similar technologies or new applications. Tactile sensors can be integrated in multimodal human-machine interfaces. Tactile images or dynamic patterns can be a way to communicate commands to a robot [8]. For instance, different gestures as “grab”, “poke”, “press”, etc. are identified by the machine through processing of the tactile map in [9]. Reference [10] presents also results of static and dynamic tactile gesture recognition for intuitive human-robot interfacing. Touch gestures such as “back left, back right, side left, side right, front left and front right” are chosen in [11] to illustrate the feasibility of tactile intuitive communication with a humanoid robot. All these works commonly extract features from the tactile map and use a classifier to determine the kind of gesture. This is also done in [12] to build isometric joysticks that are advantageous for people that suffer from tremor. This device has similarities with that proposed by the authors in [7], since it is intended to replace a conventional joystick, it does not move, and it is based on the interaction with a tactile sensor. Another similar device was also presented in [13] as steering device. When compared with other kind of sensors that could also be used to build the interface, such as cameras or force sensors [1], tactile sensors do not have the problem of occlusion that could affect to camera-based interfaces [14,15], and they can be integrated more effectively in an ergonomic interface than force sensors because they are actually a sort of artificial skin or sensitive cover [8]. However, the sensors should be conformable to adapt to different shapes, although they do not have to be necessarily stretchable since we contemplate segments or structures without moveable joints. Different technologies to implement flexible and conformable tactile sensors can be found in [16]. Advanced technologies are based on woven-like fabrics or printed electronics on flexible substrates. However, a common solution consists in using flexible Printed Circuit Boards (PCB) and solder off-the-shelf sensors to them. This is the solution implemented in this paper. A similar approach for a larger array was reported by the authors in [17]. However, this solution is only valid to conform to cylindrical shapes. To conform to other 3D surfaces it is possible to use triangular tactile sensing modules. Therefore, round fixed surfaces can hold the sensor, and many already developed systems and machines could be equipped with these devices without a costly re-design. Finally, as mentioned before, tactile sensors offer a way to communicate a large set of gestures that can enrich the capability of the interface. Nevertheless, the output of a tactile sensor is not so crisp as that from a force sensor at the time to build an interface. Firstly, the output is not a short vector, but a quite long array of data provided by all the tactile elements (tactels). Therefore, the engineer has to find the way to obtain the variables for his or her application from such output. Secondly, touch has an implicit nature of close contact with the human being, and this involves ergonomic issues that concern drastically the performance of the interface. This can enclose aspects such as different average forces for the same gesture, different ways to grasp, etc. Moreover, a transient phase could also be contemplated until the human-sensor interface is ready to clearly detect different patterns, which is observed in the case of the steering application. All these not obvious aspects about the usage and design of this interface are discussed in this paper, where results from several experiments are used to show the design rules behind.

The structure of this paper is the following: in Section 2 a brief outline of previous published work is given. It aims to help the reader better follow this article. In Section 3, the basic setup that is common to all the experiments is introduced together with a brief description of the latter and the involved parameters. Section 4 describes the interaction between the handlebar and the user in terms of force and torque and the proposal of two variables that may relate to the latter signals. Sections from Section 5, Section 6 and Section 7 cover different human factors that may influence the performance of the system based on the two suggested variables. Concretely, Section 5 explores the impact of the handlebar gripping force. In Section 6, the consequences of varying the arrangement of the tactels inside the tactile sensor are studied. In Section 7 the analysis of the grasping process is detailed. Finally, Section 8 gathers the conclusions of the present work.

## 2. Background

In this section, some previous published works that are related to the present article will be briefly summarized.

The tactile handlebar was firstly introduced as an assistive driving device aimed at wheelchair attendants in [7]. In that work it was presented, mainly from a technological standpoint, assisting the driving of a powered wheelchair (PW). In addition, a testing algorithm was proposed to perform some preliminary trials. It was based on the evolution of center of mass, CoM, computed for each handle. Tactile sensors provide maps of pressure that are normally processed to obtain high levels parameters that summarize the whole data set. The calculation of the center of mass (also known as center of pressure or centroid) is a common way to process tactile images in robotic manipulation tasks [18,19]. It concentrates the data from the tactile image in a single spatial coordinate that provides information about the pressure distribution and it was computed as explained in [7] for a tactile sensor of N×M tactels. High level parameters as the CoM are robust against typical errors present in tactile sensors such as hysteresis or drift [19]. Moreover, since the CoM depends on the pressure distribution and not directly on the values read by the tactels, it is little sensitive to pressure offset changes. That makes it be appropriate to process the pressure input of different users, that may exert distinct forces on the handlebar while driving. The use of absolute variables such as the gripping force does not provide good results as will be shown at the end of Section 4.

**Figure 1:**
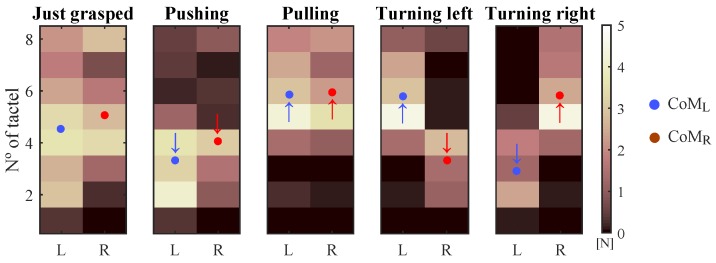
Pressure maps from the left and right tactile sensors with their corresponding centers of mass in situations in which the handlebar is: grasped at rest, pushed, pulled, turned left and right. The arrows indicate the direction of the movements of both CoM with respect to the initial situation at rest. The tactel physical location can be seen in Figure 4c.

In [7], it was noted that the centers of mass of the left and right handles changed following certain patterns when different maneuvers are carried out. As Figure 1 depicts, these parameters decrease their value when pushing and they increase it when pulling the handlebar. Besides, the CoM of each handle evolves in an opposite way when turns are performed. These findings were used to develop an algorithm. It computed the linear and angular velocities based on the movements of each handle CoM with respect to their position with no maneuver was performed. Although this algorithm was useful to show the potential of the device, it turned out to be not totally intuitive. The control output related to the linear velocity depended on the integral of the CoM deviation. This way, the linear velocity increased and decreased when the handlebar was pushed and pulled, respectively, and it remained constant otherwise. This behavior made that attendants needed to be instructed to use the device properly, since it did not work like a conventional handlebar. The user previous experience was not being exploited, which limited the effectiveness of the tactile handlebar as driving interface.

**Figure 2:**
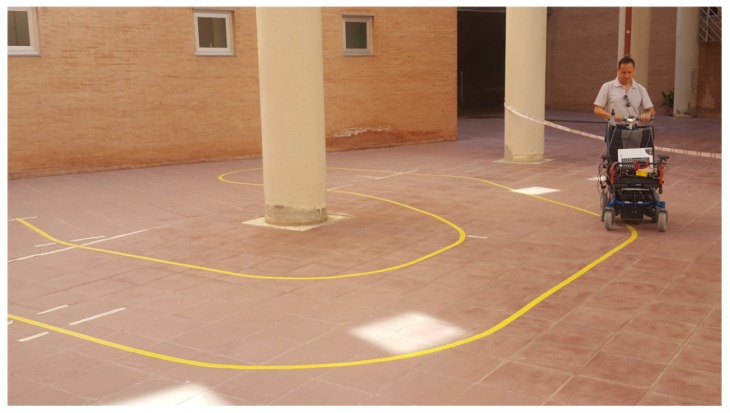
Participant performing the experiment presented in [3].

Furthermore, a controlled experiment was undertaken in the work presented in [3]. It assessed the tactile handlebar as a driving interface in an open and wide space as well as in a narrow environment with obstacles (Figure 2 shows the performing of one of the tests included in [3]). In addition, the perception and opinion of the participants were analyzed. The tactile handlebar was also compared with the attendant joystick, which is the market standard aimed to assist this kind of users. The results obtained in [3] were good in general terms and this was possible due to the improvement of the user intention detection proposed in [7]. A deeper analysis of the system and the identification of several key points that were pivotal elements in the experiment realizations of [3] will be presented in this article.

## 3. Experimental Setup and Parameters of Interest

A set of experiments will be introduced throughout the document. All of them are based on the same experimental setup, to a greater or lesser extent. It involves, basically, the assistive handlebar, its conditioning electronics and a computer that gathers the captured data for their analysis. Some additional elements are used depending on each specific experiment. The ambulatory device that has been chosen to test the handlebar is a powered wheelchair.

**Figure 3:**
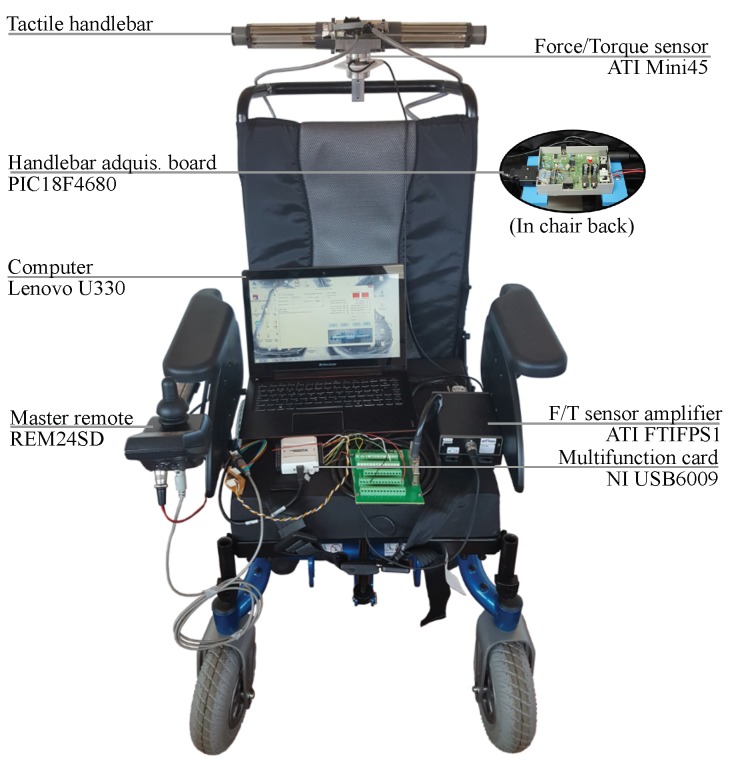
Experimental setup designed to perform the different experiments of this article.

Figure 3 shows the complete experimental setup. It includes a Force/Torque Mini45 sensor (ATI Industrial Automation, Apex, NC, USA) located between the handlebar and the wheelchair. It obtains ground truth measurements of force and torque applied on the handlebar. Both signals are processed by the amplifier ATI FTIFPS1 (ATI Industrial Automation, Apex, NC, USA) and then digitized by the multifunction card NI USB6009 (National Instruments, Austin, TX, USA). At the same time, the pressure on the tactile handlebar is read by the tactile sensor that each handle incorporates and processed by the conditioning electronics designed for this purpose. It is based on a PIC18F4680 microcontroller (Microchip Technology Inc., Chandler, AZ, USA) and sends the pressure data to a computer via USART-USB. This electronics was explained at length in our previous work in [7]. The computer (Lenovo U330, Lenovo Group Ltd., Hong Kong, China) is in charge of gathering the data of force, torque and handlebar pressure synchronously at a rate of 60 Hz.

**Figure 4:**
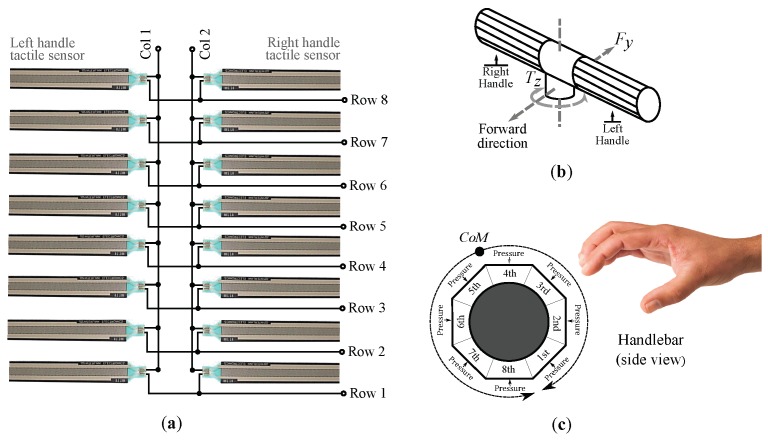
Tactile handlebar: (**a**) Schematic of tactile sensors. (**b**) Force and torque involved in the driving of a PW through the handlebar. (**c**) Handlebar tactel arrangement and the CoM range of movement.

The powered wheelchair of Figure 3 (Bora, Invacare, Elyria, OH, USA) is a heavy ambulatory device and it is not designed to be manually propelled. Its weight is equivalent to that of a conventional wheelchair with person around 80 kg seated. In those tests in which the PW is moved, the driving is assisted by the engines of the wheels. On the other hand, the tactile handlebar is composed of a matrix of 8×2 tactels (see Figure 4a). This kind of configuration minimizes the required addressing resources of the acquisition electronics. Each tactel is formed by a force sensor resistor (FSR) shaped like a rectangular strip (FSR408${}^{®}$, Interlinks Electronics, Camarillo, CA, USA). The operating principle of these sensors is based on the piezoresistive effect. Hence they behave as variable resistors whose value changes according to the exerted pressure. Please note that although the two columns of the matrix are electrically interconnected they are processed as two different linear tactile sensors, one for the left and other for the right handle.

Two main parameters have been used along this work. The remaining variables are based on them. One is the center of mass computed for the right and left handle, $CoM_{L}$ and $CoM_{R}$, that was introduced in the previous section. For a linear array as the tactile sensor of each handle (see Figure 4a), it is computed as:

(1) $$\begin{array}{c} CoM=\frac{\sum_{y=1}^{8}y\cdot p\left(y\right)}{\sum_{y=1}^{8}p\left(y\right)} \end{array}$$

where *y* and p(y) are the position and the pressure value of the *y*th tactel in the handle for which the CoM is being calculated (see Figure 4c). The other is the gripping force, GF. It is the force exerted by the attendant when grasping the handles. One way to estimate it as a single value could be to compute the mean force on the tactile handlebar when it is grasped:

(2) $$\begin{array}{c} GF=\frac{\sum_{x=1}^{2}\sum_{y=1}^{8}f\left(x,y\right)}{16} \end{array}$$

where f(x,y) is the force on the tactel located at coordinate (x,y). Please note that the coordinate points are referred to the rows and columns of tactile matrix of Figure 4a.

The experiments that were carried out have to do with the previous parameters and have the following purpose:

- **EA**: Experiment aimed to identify CoM-based control inputs capable of predicting the user intention.
- **EB**: Experiment aimed to analyze the influence of the gripping force on the control inputs when grasping the handlebar.
- **EC**: Experiment carried out with the purpose of studying how the tactel configuration inside the tactile array affects the proposed control inputs.
- **ED**: Experiment conducted to study the grasping process in terms of CoM evolution. Some aspects as the impact of the user height or the gripping force on this process are also studied.

In the following sections, these experiments together with their results will be explained and discussed.

## 4. Tactile Control Inputs Based on Force/Torque and Pressure Analysis

The main aim of this section is to explore how the information extracted from the tactile handlebar may be processed, in order to achieve that our device can be operated as similar as possible as a regular handlebar. With that purpose an experiment (**EA**) was designed. It was aimed to obtain and analyze the data involved in the PW driving.

The first step involved the description of the interaction between the attendant and the wheelchair handlebar. To this end, maneuvering can be distinguished between push/pull and turns. The driving of a wheelchair by an attendant can be described in terms of this kind of maneuvers and their combinations. Pushing/pulling maneuvers can be modeled as a force vector, $F_{y}$, in the plane parallel to the ground, in the walking direction (see Figure 4b). Furthermore, the turning resistance may be seen as the torque, $T_{z}$, needed to turn the wheelchair in its smallest circle, whose center is in the halfway between the two fixed rear wheels [20]. The handlebar center is located at approximately the same point, in a higher parallel plane x−y. This way, $F_{y}$ and $T_{z}$ may be used to identify and quantify pushing/pulling maneuvers and turns (this approach is also used, for example, by the authors of [1] to design the control of a robotic supermarket trolley).

The authors hypothesized that two variables computed using the center of mass provided by the left and right tactile sensors, $CoM_{L}$ and $CoM_{R}$, were linked to the force and torque exerted on the handles by the attendants. They are the sum and the subtraction of the CoM of each handle, $SUM_{CoM}$ and $SUB_{CoM}$. These variables are calculated as:

(3) $$\begin{array}{c} SUM_{CoM}=CoM_{L}+CoM_{R}\\ SUB_{CoM}=CoM_{L}-CoM_{R} \end{array}$$

Specifically, it was conjectured that there was a coupling between $SUM_{CoM}$ and $F_{y}$, that is to say, the force required to move forward or backward the PW, and between $SUB_{CoM}$ and $T_{z}$, the signal that represents the torque exerted in turns. This assumption is in line with intuition by observing the shifts of $CoM_{L}$ and $CoM_{R}$ while different maneuvers are carried out. Figure 1 is useful again to understand the proposal. Firstly, the attendant just grasps the handlebar without exerting intentional forces. Thereafter, he or she pushes and pulls it. Please note that in these cases, both CoM move in the same direction, so their sum decreases or increases depending on whether the handlebar has been pushed or pulled. $F_{y}$ is a signal that also varies when this kind of maneuvers are exerted (see Figure 4b). Regarding left and right turns, both CoM move in opposite directions and, thus it is their subtraction what increases or decreases. In the same way, $T_{z}$ changes when turning the handlebar (see Figure 4b).

In the next section, details of the experiment aimed to assess the feasibility of the proposed variables are provided.

### 4.1. Methods

**Figure 5:**
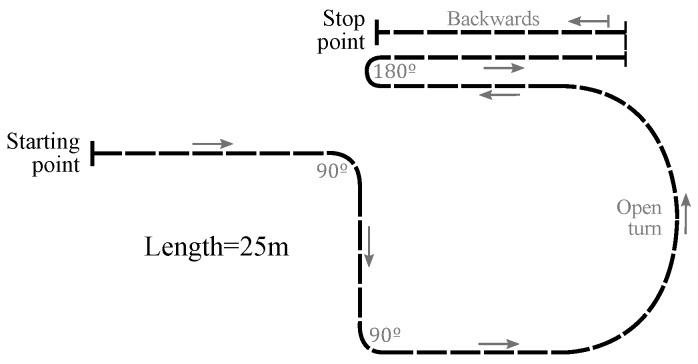
Path of the experiment **EA**.

Ten volunteers (**PA1**–**PA10**) with no previous experience in wheelchair driving and without any movement disorders took part in the test. They were between 24 and 63 years old, with an average age of 39.6. Given writing consent and complying with the ethical principles of the declaration of Helsinki at all times, they used the experimental setup of Section 3 to drive the wheelchair through handlebar along the path showed in Figure 5. Marks on the ground and plastic cones were used to help the participants to follow the trajectory. The path gathered, for about 25 m, the typical maneuvers present in the normal usage of a handlebar. These were: several forward movements, two 90° turns, an open turn, a 180° turn around and a backward movement. Around one minute is required to cover the path length. The participants were not aware of the experiment purpose. Two trials were carried out. The first one was used to familiarize the participants with the system and the path. During the second test $F_{y}$, $T_{z}$ and the tactile output were registered as explained previously. The volunteers did not receive instructions about how to drive the PW with the device, so that their performance was based on their previous experience using handlebars.

The wheelchair driving was assisted by the engines in the wheels during the tests. The activation of the engines was carried out in an indirect way, by emulating a series of analog signals, $V_{v}$ and $V_{\omega }$, provided by the joystick that the PW includes [7]. $F_{y}$ and $T_{z}$ were used as control signals to compute the linear and angular velocities, *v* and ω (see Equation (4)). Please note that, as explained above, $F_{y}$ is related to forward/backward movement and therefore to linear speed, and $T_{z}$ to turns and angular speed.

(4) $$\begin{array}{c} V_{v}=G_{lin}F_{y}\\ V_{\omega }=G_{ang}T_{z} \end{array}$$

where $V_{v}$ and $V_{\omega }$ are the joystick output voltages that control the linear and angular velocities, and $G_{lin}$ and $G_{ang}$ are gains to adapt the maneuvering sensibility to the user preferences.

The potential link between on the one hand, $SUM_{CoM}$ and $F_{y}$, and on the other, $SUMB_{CoM}$ and $T_{z}$, was evaluated by computing the Pearson correlation coefficient, *r*, for the tests carried out in the experiment. It measures the linear relationship between two variables. To assess the size of the correlation coefficients, the rule of thumb proposed by Hinkle et al. [21] is used (see Table 1).

The four parameters involved in the analysis were low-pass filtered to remove possible noise and interferences. Besides, the initial and the final captured samples in which both centers of mass are not stable were discarded. These correspond to the very first moment in which the participant grasps the handlebar and to the instant when the user releases it at the end of test.

### 4.2. Results and Discussion

The correlation coefficients obtained for $SUM_{CoM}$ and $F_{y}$ and $SUB_{CoM}$ and $T_{z}$ are listed in Table 2. As can be observed, for the former two variables the correlation was high positive for nine participants and moderate positive for one of them. Regarding the latter, it was practically high positive for all the participants. Moreover, it was statistically significant in all the tests (with a significance threshold p=0.05).

**Figure 6:**
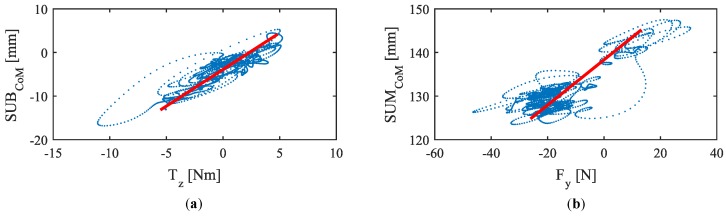
Coupling between the variables (**a**) <$SUB_{CoM}$,$T_{z}$> and (**b**) <$SUM_{CoM}$,$F_{y}$> with the 1st order approximations superimposed for one participant of the experiment **EA**.

Figure 6 illustrates one of the variables versus the other (with the 1st order approximation superimposed) for an average participant of the experiment **EA**. In the case of $SUM_{CoM}$ and $F_{y}$ (Figure 6b), the bulk of the samples are mainly concentrated at the bottom of the graph. Please note that during the major part of the experiment, the PW is moving through the path with positive linear speed that is produced by pushing the handlebar. When pushing, both CoM move to the lower tactels of the tactile arrays (rear part of the handles, see Figure 4c) so that their values are minimum. Consequently, the parameter $SUM_{CoM}$ will be also minimum. Please note that for $SUB_{CoM}$ and $T_{z}$, the higher point density is located at the central area of the graphs. This part corresponds to the absence of turns. Turns are temporary maneuvers during which samples are shifted from this area to one of the extremes of the chart, depending on whether the turn is to the left or to the right. Most of the time PW goes straight so it is foreseeable that the points are concentrated in the central area.

**Figure 7:**
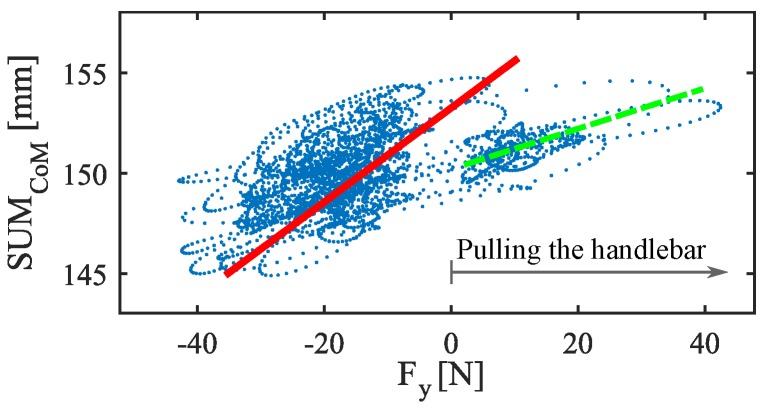
$SUM_{CoM}$ versus $F_{y}$ for **PA7**. The red line is computed from the data of **PA7** by linear regression. The green dashed line, which has a lower slope, fits better with the data captured during pulling maneuvers.

Although the results are generally robust, the seventh participant (**PA7**) presents a correlation for <$SUM_{CoM}$,$F_{y}$> that, despite not being weak, is a little lower than for the others. It seems that, in his case, there is an asymmetry between pushing and pulling maneuvering. Figure 7 helps visualize the mismatching. Please note that the first order function that fits in the test carried out by **PA7** deviates slightly from the data captured while pulling the handlebar, which affects to the linear relationship between the variables. This effect has not been observed so clearly in the rest of the tests. Furthermore, it is also interesting to comment that sometimes the PW experienced some tugging while driving. These little sudden movements seemed to be modulated by the participant gait.

Notwithstanding the above, it should be borne in mind that the experiment was carried out by persons who present physical and behavioral differences. A range of parameters may influence the correlation: the fact of comparing outputs from sensors of different kind, the gripping force or the way of grasping the handlebar, among others that will be addressed in next sections. Despite the case of moderate positive correlation of **PA7**, the presented results are good enough to show that the substitution of the control inputs used to compute the wheelchair movement by those obtained from the tactile handlebar seems to be viable. Specifically, $SUM_{CoM}$ detects and quantifies push and pull maneuvers and $SUB_{CoM}$ does the same with turns.

Other variables can be used as control inputs. That is the case, for example, of the work presented in [13]. Its authors used tactile sensors to estimate the user intention in a walking support robot using a bicycle-type handlebar. As with our proposal, they covered the left and right handles with FSR sensors obtaining two tactile sensors, each of 3×8 tactels. They propose the subtraction of the gripping force on each handle, ($GF_{L}$ − $GF_{R}$), as a variable to control turns. The results of the experiment that they performed show a high correlation between this variable and the measurements of a F/T sensor (r=0.91 for the best performance of five trials).

**Figure 8:**
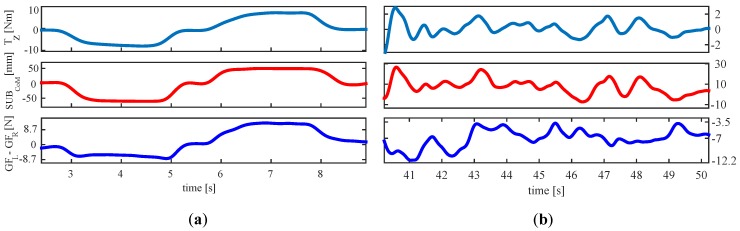
(**a**) Results of the replication of the test performed in [13] and (**b**) results of applying the idea proposed in [13] in a test of the experiment **EA**. From top to bottom: signal captured by a F/T sensor, parameter proposed in this work and parameter proposed in [13].

Since this approach is closely related to that presented in this article, it was worth to replicate it using the tactile handlebar, involving as well a turn to the right and another to the left. The result is shown in Figure 8a. As can be observed, both proposals provide signals that follow closely that measured by the F/T sensor. The link was assessed with Pearson and Spearman’s rank order correlation. The latter, ρ, provides a measure of how monotonic is the relationship between two variables. For $<GF_{L}-GF_{R},T_{z}>$, the coefficients were: r=0.87 and ρ=0.85. For $<SUB_{CoM},T_{z}>$, they were: r=0.98 and ρ=0.99. Both parameters show an strong link that could be use to detect turning intentions.

On the other hand, the experiment realized in [13] represented an ideal situation. The use of a walking support robot involves not only turns, but also forward and backward maneuvers and combinations of both. This scenario was reflected in the experiment **EA**. The data acquired in it were processed to assess the link of $GF_{L}-GF_{R}$ and $T_{z}$ in a more realistic situation. This way, the Pearson correlation coefficients for $<GF_{L}-GF_{R},T_{z}>$ were: r=−0.26[**PA1**], r=−0.17[**PA2**], r=0.26[**PA3**], r=−0.07[**PA4**], r=0.11[**PA5**], r=−0.36[**PA6**], r=0.27[**PA7**], r=−0.03[**PA8**], r=−0.12[**PA9**] and r=0.12[**PA10**]. As observed, the results were not good. Correlations were low or directly negligible. It seems that, when steering does not involve exclusively turns, $GF_{L}-GF_{R}$ is no longer a good turning intention predictor. This is clearly illustrated in Figure 8 right. Whereas $SUB_{CoM}$ (center) follows the changes of the signal captured by the F/T sensor (top), $GF_{L}-GF_{R}$ (bottom) does not appear to relate to the previous ground truth measurement.

## 5. Study of the Gripping Force Influence

As anticipated in Section 3, the gripping force is that exerted by the attendant when grasping the handlebar. It is a relevant parameter for the device usage, due to the nature of the used sensors. Different kind of grips may have distinct characteristics and affect the pressure maps in different ways. Therefore, an analysis of the gripping force may provide interesting information that helps design the driving control.

### 5.1. Grip Force Impact on the Link between Force and Torque Involved in Driving and the Parameters Obtained by the Tactile Handlebar

In the previous section, two variables that identify the user intention when pushing, pulling and turning the handlebar were presented. They were highly correlated with the force and torque involved in the driving.

**Figure 9:**
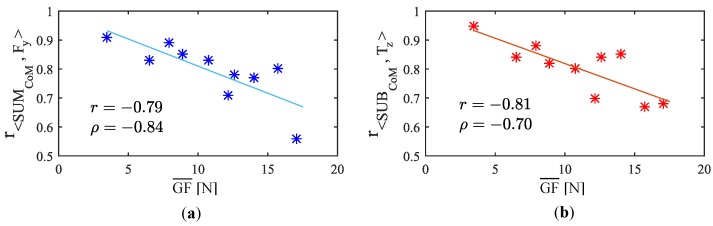
GF effect on the links between (**a**) <$SUM_{CoM},F_{y}$> and (**b**) <$SUB_{CoM},T_{z}$> (1st order functions superimposed).

A first step to assess the influence of the GF on the driving control is to check how it affects the capacity of the proposed variables to identify the user intention. With this purpose, the mean gripping force, $\bar{GF}$, exerted during the tests of the experiment **EA** was calculated. Figure 9 relates the correlation coefficient obtained for each test of the experiment **EA** (listed in Table 2) to the mean GF exerted during the trial.

As can be clearly seen, the correlation between the proposed variables and $F_{y}$ and $T_{z}$ decreases as the gripping force increases. This tendency has been assessed with Pearson and Spearman’s rank-order correlations. The coefficients are r=−0.79(p=0.0064) and ρ=−0.84(p=0.0024) for the link between the coupling <$SUM_{CoM}$,$F_{y}$> and the gripping force, and r=−0.81(p=0.0048) and ρ=−0.70(p=0.03) for that between the coupling <$SUB_{CoM}$,$T_{z}$> and the gripping force. These values imply a high negative correlation of both types Pearson and Spearman’s rank-order. It reveals that higher gripping forces would presumably lead to a worse control of the device and, therefore, to a poorer driving experience.

### 5.2. Grip Force Impact on the Excursion of the Centers of Mass

In some preliminary tests, it was observed that when the handles were grasped too strongly, the range of variation of the proposed variables, $SUM_{CoM}$ and $SUB_{CoM}$, decreased significantly. This phenomenon was likely to be related to a reduction of the excursion of the centers of mass. The CoM excursion could be defined as the maximum distance that the CoM covers through the tactile sensor while using the tactile handlebar.

**Figure 10:**
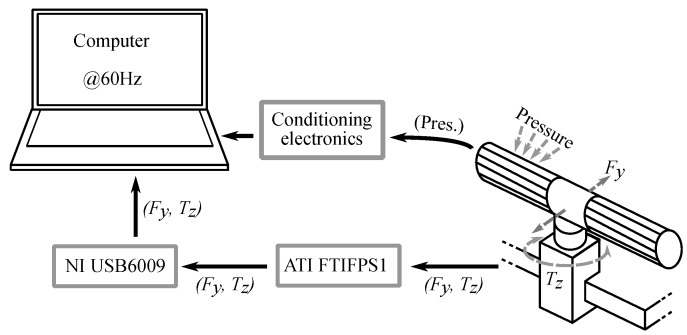
Experimental setup scheme of the experiment **EB**. The handlebar is fixed to a laboratory table.

An experiment (**EB**) was designed to analyze if the gripping force affects the CoM excursion and limits it somehow. Figure 10 shows the experimental setup scheme. The only difference with respect to the setup of Figure 3 is that the handlebar was removed from the PW and fixed to a support clamped to a laboratory table. Pressure data from tactels and $F_{y}$ and $T_{z}$ from the F/T sensor were gathered by the computer at 60 Hz.

#### 5.2.1. Methods

**Figure 11:**
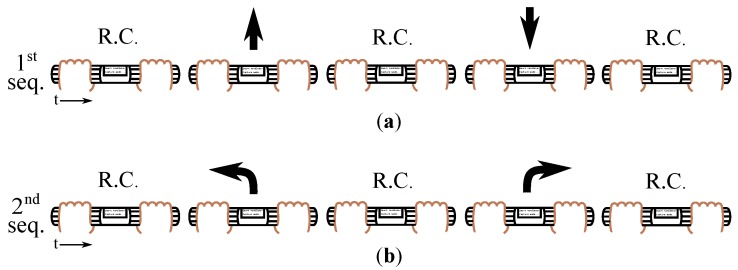
(**a**) During the 1st sequence, pushing and pulling maneuvers were exerted. (**b**) In the 2nd sequence, turns were performed.

Seven volunteers (**PB1**–**PB7**) from 21 to 32 with a mean age of 26.9 years took part in this study after agreement and informed consent. They were given instructions to realize a set of maneuvers (see Figure 11). Firstly, they grasped the handlebar and performed the following sequence:

(1) Rest condition (it consists in just keeping the handles grasped without exerting intentionally forces) (R.C.)→push→rest condition→pull→rest condition. They had to keep the current condition (push, rest or pull) at least for one second before changing to the next state. After this first test, they were asked to carry out a new sequence:
(2) Rest condition→left turn→rest condition→right turn→rest condition.

The described sequences were repeated three times, each with a different gripping force: grasping the handlebar weakly, normally (They grasped the handles in a way they considered normal or natural.) and strongly. “Weakly”, “normally” and “strongly” are subjective terms and, e.g., what is a weak grasp for a user can be a strong one for another. The purpose of giving the participants these commands was to ensure the availability of the maneuvers carried out with different gripping forces by the same person, in the same conditions. Considering the three kinds of grips for both types of maneuvers, 42 tests were undergone, 21 for pushing/pulling maneuvering and another 21 for turns. All of them were low-pass filtered to reduce noise.

#### 5.2.2. Results and Discussion

**Figure 12:**
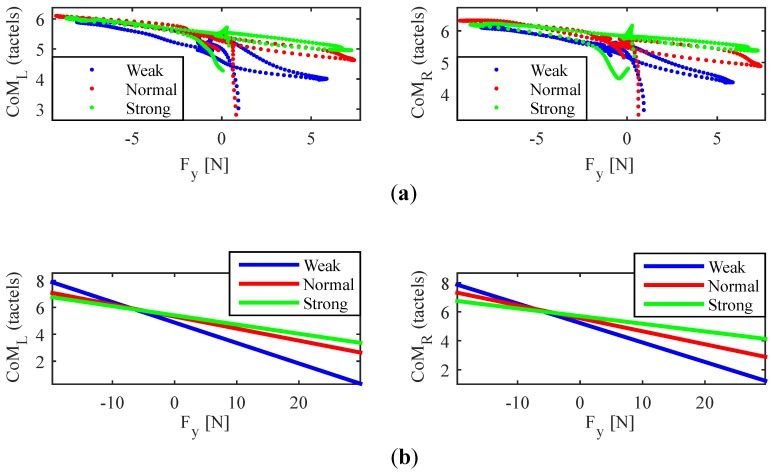
(**a**) $CoM_{L}$ and $CoM_{R}$ versus $F_{y}$ during pushing and pulling maneuvers (1st sequence) for an average participant of the experiment **EB**. (**b**) Linear approximations.

**Figure 13:**
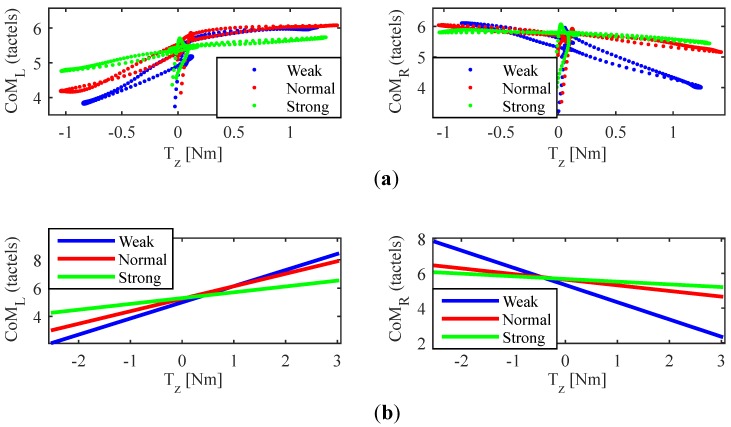
(**a**) $CoM_{L}$ and $CoM_{R}$ versus $T_{z}$ during the turns (2nd sequence) for an average participant of the experiment **EB**. (**b**) Linear approximations.

Figure 12 and Figure 13 show the data collected for an average participant of the experiment **EB** during the sequence of push/pull maneuvers (1st) and that of turns (2nd), respectively.

Please note that there are four graphs in each figure. In the case of Figure 12, the upper two represent in *y*-axis the variation of the center of mass of each handle (location expressed in tactel coordinates), $CoM_{L}$ and $CoM_{R}$, with respect to $F_{y}$, in *x*-axis. The lower two are the linear approximation of these two functions. Regarding Figure 13, *y*-axis gathers the same information and *x*-axis displayed $T_{z}$, which is the variable of interest when turning. As can be observed in both figures, the linear approximations present a slope that clearly decreases as the grip passes from “weak” to “normal” and from “normal” to “strong”, both for the pushing/pulling maneuvering and for the turn sequences. This is a phenomenon that affects all the tests performed by the participants so that it suggests that the higher the gripping force, the lower the CoM excursion is. An analysis of the GF evolution and the tactels response could throw some light on the causes behind this effect.

**Figure 14:**
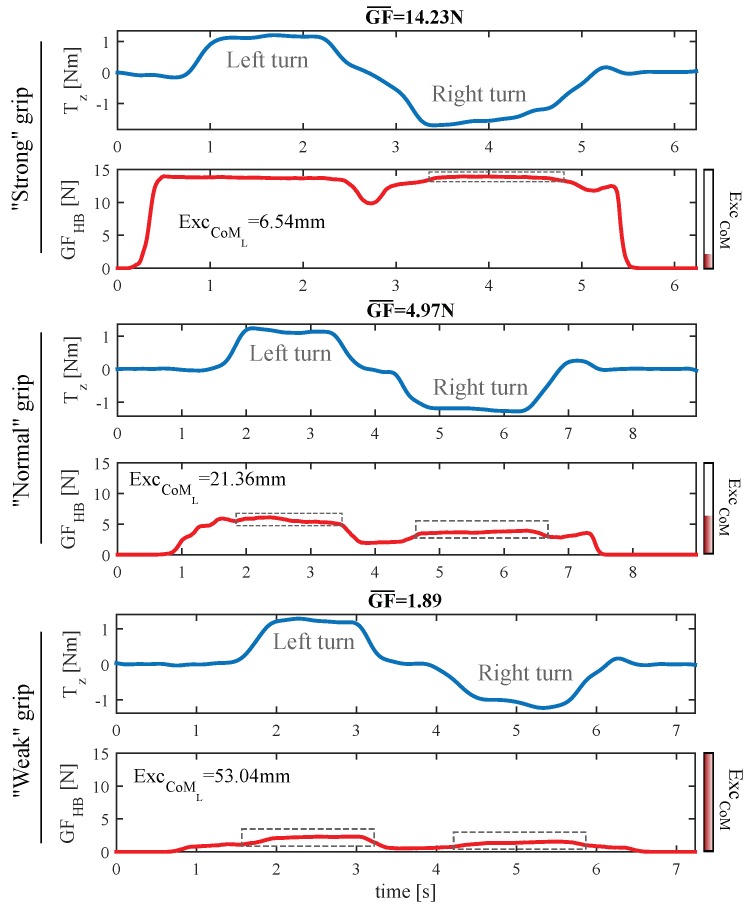
From top to bottom: $T_{z}$ and GF on the handlebar during the performance of the sequence of turns of the experiment **EB** by a participant of the experiment **EB** for “strong”, “normal” and “weak” grip.

Figure 14 illustrates the changes in the gripping force shape as it increases. The lower two charts display $T_{z}$ (in blue) and the GF on the handlebar ($GF_{HB}$, in red) and they belong to the sequence of turns of one participant who was applying a “weak” grip. The GF variation due to the left and right turns is well visible just by comparing the changes of GF (dashed rectangles) and the ATI Mini45 torque output when performing the maneuvers. In the two central graphs, those corresponding to the “normal” grip test of the same subject, the influence of the turn maneuvers on GF is still perceptible, although the “offset” caused by the grasp is higher. Finally, the two uppers correspond to the performance of the same sequence, this time with a “strong” grip. As can be seen, GF hardly varies; it gets saturated a considerable part of the time and is almost unaffected by the turns. As shown in the figure, the $CoM_{L}$ excursion has gone down from 53.04 mm to 21.36 mm (“weak” to “normal”), and from 21.36 mm to 6.54 mm (“normal” to “strong”) as the GF increased. This is a reduction of 59.7% and 87.7%, respectively. This results were similar for the $CoM_{R}$.

A direct cause behind the loss of the CoM excursion may be the tactels saturation. In fact, some of the tactels got saturated in the “strong” grip test of Figure 14. However, this seems not to be the only reason. In the case of the “normal” test of the same figure, the maximum force values registered by the tactels were $\left[f_{L1_{MAX}},f_{L2_{MAX}},\ldots ,f_{L8_{MAX}}\right]=\left[2.65,5.73,2.74,11.48,10.03,3.85,8.02,4.34\right]$*N* for the left handle and $\left[f_{R1_{MAX}},f_{R2_{MAX}},\ldots ,f_{R8_{MAX}}\right]=\left[7.2,10.76,0.91,13.04,9.68,3.81,11.76,4.66\right]N$ for the right one. A characterization of the tactels was performed to determine the saturation threshold. It was found that their output was linear way up to 18N, value above which became saturated. According to previous numbers, the tactels were far from saturation for the “normal” test. Still, the CoM excursion experienced a fall of 59.7% with respect to that of the “weak” grip. The only difference between both tests is the handlebar gripping force. What the data seem to show is that the lack of excursion could also have an anatomic nature: as hands increase the gripping force, they would experience a growing loss of their capacity to introduce pressure variations.

#### 5.2.3. Correction of the Gripping Force Impact on *CoMs* Excursion

**Figure 15:**
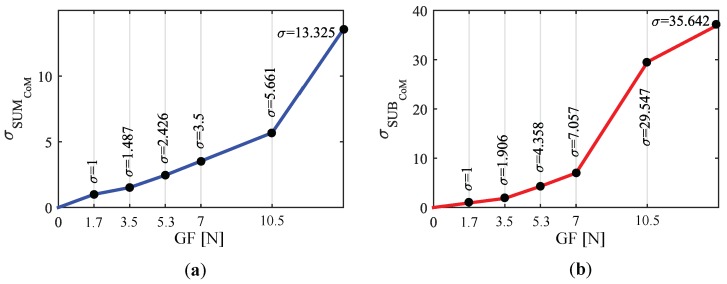
Gripping force-dependent gain functions for (**a**) $SUM_{CoM}$ and (**b**) $SUB_{CoM}$.

The direct consequence of the effect explained above is that the output of the system may be affected by the GF in terms of the powered wheelchair linear and angular speeds. Attendants who tend to grasp stronger will need to push, pull or turn stronger than those who grasp weaker in order to get the same CoM excursion and, therefore, the same output and speed. A way to proceed to minimize this effect consists in making the output gain dependent of the GF. The data of the experiment **EB** were used to build the variable gain curves of Figure 15. It shows two variable gain functions, $\sigma_{SUM_{CoM}}$ and $\sigma_{SUB_{CoM}}$, one of them for $SUM_{CoM}$ and the other for $SUB_{CoM}$. When a new pressure map is received and the control inputs are computed, their slope, $m_{SUM_{CoM}}$ and $m_{SUB_{CoM}}$, is corrected according to the gripping force. It minimizes the detrimental effect of the latter:

(5) $$\begin{array}{c} m_{SUM_{CoM_{corrected}}}=\sigma_{SUM_{CoM}}\cdot m_{SUM_{CoM}}\\ m_{SUB_{CoM_{corrected}}}=\sigma_{SUB_{CoM}}\cdot m_{SUB_{CoM}} \end{array}$$

The functions of Figure 15 were calculated as follows: all the tests belonging to the 1st and 2nd sequences (see Figure 11) were classified into six groups according to the exerted mean gripping force. These groups were $G_{PP_{1}}\ldots G_{PP_{6}}$ for the tests belonging to the 1st (pushing/pulling) and $G_{T_{1}}\ldots G_{T_{6}}$ for those from the 2nd sequence (turns). The chosen intervals were: $0<\bar{GF}\leq 1.7\;N\left(G_{\mathrm{PP},T_{1}}\right)$, $1.7<\bar{GF}\leq 3.5\;N\left(G_{\mathrm{PP},T_{2}}\right)$, $3.5<\bar{GF}\leq 5.3\;N\left(G_{\mathrm{PP},T_{3}}\right)$, $5.3<\bar{GF}\leq 7\;N\left(G_{\mathrm{PP},T_{4}}\right)$, $7<\bar{GF}\leq 10.5\;N\left(G_{\mathrm{PP},T_{5}}\right)$, $10.5\;N<\bar{\mathrm{GF}}\left(G_{\mathrm{PP},T_{6}}\right)$. The interval limits were adjusted taking into account the variability of the $\bar{GF}$ exerted in the different tests and trying to have a similar number of them in each group.

**Figure 16:**
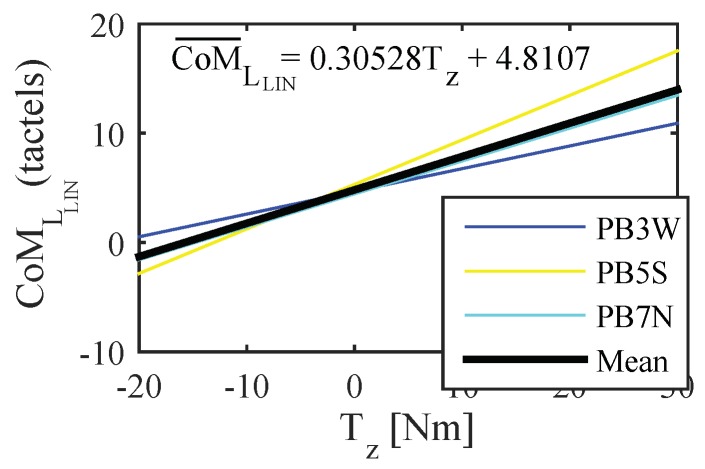
Linear approximation $CoM_{L_{lin}}$ of the tests included in the group $G_{T_{3}}$ and mean of these functions (thicker line in black). The tests within this group ($3.5<\bar{GF}\leq 5.3$ N) where those from the participants **PB3** (‘weak’), **PB5** (‘strong’) and **PB7** (‘normal’).

The linear approximation, $CoM_{LIN}$, was computed for every test of the experiment **EB**. Figure 16 shows the linear approximation of left handle CoM computed for the tests belonging to the group $G_{T_{3}}$ ($\bar{GF}$ between 3.5 and 5.3 N).

The mean function of the linear approximations of each group, ${\bar{CoM}}_{LIN}$, was computed as expressed in Equation (6). In Figure 16, this function can be seen in a thicker black line for one of the groups.

(6) $$\begin{array}{c} {\bar{CoM}}_{H_{LIN}}=\frac{\sum_{i=1}^{N}CoM_{H_{LIN_{i}}}}{N}=X\frac{\sum_{i=1}^{N}a_{H_{S_{i}}}}{N}+\frac{\sum_{i=1}^{N}b_{H_{S_{i}}}}{N}={\bar{a}}_{H_{S}}X+{\bar{b}}_{H_{S}} \end{array}$$

where $\bar{a}$ and $\bar{b}$ are the slope and zero of the mean linear function and the other terms and subscripts have the following meaning:
*N*Number of tests inside the group ($G_{PP,T_{1}}\ldots G_{PP,T_{6}}$) for which ${\bar{CoM}}_{H_{LIN}}$ is calculated*i*Each of tests of the group for which ${\bar{CoM}}_{H_{LIN}}$ is calculated*X*Signal that varies in the group for which the function is computed: ***F_y_*** for $G_{PP_{1}}\ldots G_{PP_{6}}$ and ***T_z_*** for $G_{T_{1}}\ldots G_{T_{6}}$*S*Sequence the test *i* belongs to: ***PP*** for the tests in $G_{PP_{1}}\ldots G_{PP_{6}}$ and ***T*** for those in $G_{T_{1}}\ldots G_{T_{6}}$*H*Tactile handle for which the parameter is calculated: ***L*** and ***R*** (left or right)

The gradients of the functions computed using the previous expression are listed in Table 3. They illustrate the mean rate of change of the CoM with respect to $F_{y}$ and $T_{z}$ as the gripping force increases.

Given that the control inputs depend directly on $CoM_{L}$ and $CoM_{R}$, they are affected by the excursion reduction. Using the mean of the linear approximations in (6):

(7) $$\begin{array}{c} {\bar{SUM}}_{CoM}={\bar{CoM}}_{L_{LIN}}+{\bar{CoM}}_{R_{LIN}}=\left({\bar{a}}_{L_{PP}}+{\bar{a}}_{R_{PP}}\right)F_{y}+\left({\bar{b}}_{L_{PP}}+{\bar{b}}_{R_{PP}}\right)\\ {\bar{SUB}}_{CoM}={\bar{CoM}}_{L_{LIN}}-{\bar{CoM}}_{R_{LIN}}=\left({\bar{a}}_{L_{T}}-{\bar{a}}_{R_{T}}\right)T_{z}+\left({\bar{b}}_{L_{T}}-{\bar{b}}_{R_{T}}\right) \end{array}$$

The values of Table 3 were used to build variable gain coefficients that reduce the impact of the GF. Please note that, as shown in Table 3, the groups with the highest gradients of the proposed variables, $m_{SUM_{CoM}}$ and $m_{SUB_{CoM}}$, are $G_{PP_{1}}$ and $G_{T_{1}}$. As can be seen in Figure 15, there is no amplification for their upper limit (σ=1). In addition, there is an attenuation for forces between 0 and 1.7 N, with the purpose of avoiding unwanted PW movements caused by not firm enough grips. The rest of values in Figure 15 were calculated so that the groups $G_{PP,T_{2}}$…$G_{PP,T_{6}}$ have the same gradient of the control inputs as $G_{PP,T_{1}}$ in their upper limit. Linear interpolation was chosen since it requires low computation and the result was similar to that using more complex basis functions.

In addition to that reported above, excessive gripping forces also lead to overexertion and fatigue. Another strategy implemented to prevent them consisted of adding vibro-haptic feedback to the handles. Vibrations are widely used as notification signals in haptic support systems [22]. Besides, assistive technology users have a preference for a warning systems that are inconspicuous [23,24,25], that is the case of vibration-based alerts. One DC vibration motor (C-6070 by Cebek, Barcelona, Spain), was inserted into each handle to implement the haptic feedback. This add-on was used in the experiment undertaken in [3] to warn the participants when too high forces were exerted. It contributed to the good results achieved in [3].

## 6. Study of the Effect of the Tactel Arrangement

Wider CoM excursions result in a bigger range of variation of the variables $SUM_{CoM}$ and $SUB_{CoM}$, which in turn allows assistants to have a larger control of the device. The tactel arrangement of the tactile sensor is that of Figure 4c. Let us remind that the centers of mass are calculated as indicated in Equation (1), where *y* refers to the position of each tactel. Thus, if tactels are sorted in a different way, the obtained CoM will be different even if the map of pressure remains the same. In the same way, the CoM excursions may change by choosing a different tactel spatial configuration.

An experiment (**EC**) was designed to explore the influence of changes in the array configuration on the parameters of interest. The experimental setup was that of Section 3. The wheelchair was mechanically braked and locked to prevent it from moving during the trials.

### 6.1. Methods

Twelve participants took part in the experiment after agreement and informed consent, and without knowledge of its purpose. They were between 26 and 63 years old, with a mean age of 38.6 years. They were asked to carry out twice each of the sequences presented in the experiment **EB** (see Figure 11). Moreover, they did it with the handlebar adjusted at the minimum ($h_{1}$ = 98.5 cm) and maximum ($h_{2}$ = 108 cm) height allowed by the settings of the used PW (F35 by Sunrise Medical, Fresno, CA, USA). They were not given any specification about how to grasp the handlebar. The total number of performed tests was 48 for each handlebar height (24 tests for the sequence of push/pull maneuvers and another 24 for the sequence of turns). All of them were filtered in order to reduce noise before being analyzed.

**Figure 17:**
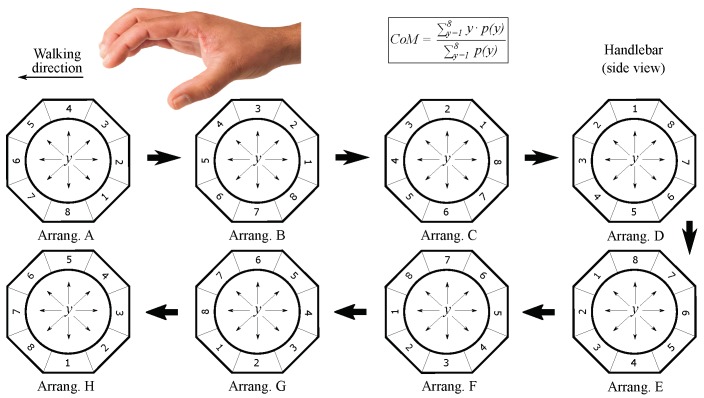
Tactel arrangements for the calculation of the CoM of the tests from the experiment **EC**.

For every trial, the CoM of the left and the right handle was computed 8 times, using the 8 tactel arrangements presented in Figure 17. The correspondence between the values of the variable *y* and the real tactel locations that they refer to can be seen in the figure. Excursion was calculated for $CoM_{A}$, $CoM_{B}$, $CoM_{C}$,…, $CoM_{H}$ (the subindex represents the tactel configuration for which the CoM was calculated).

### 6.2. Results and Discussion

As said above, excursion was calculated for each of the eight available centers of mass ($CoM_{A}$-$CoM_{H}$) in every test. The number of times for which each CoM got the largest excursion is listed in Table 4 and Table 5.

The former gathers the data of the tests carried out at a handlebar height of $h_{1}=98.5$ cm. Looking at the table, it seems clear that $CoM_{E}$ is the center of mass that achieved the largest excursion in most of the cases. Only in the sequence of turns, other center of mass obtained better results ($CoM_{F}$ in the left handle). However, in this case, $CoM_{E}$ still got a good number, being the second best option. Regarding the tests undertaken at a height of $h_{2}=108$ cm (see Table 5), the tendency is even clearer. $CoM_{E}$ produced the largest excursion in all the tests, considering both pushing and pulling maneuvering and turns. This way, it seems that the tactel arrangement *E* (see Figure 17) is that that provides the highest CoM excursion more frequently.

The obtained results suggest that arrangement *E* is a good choice from the CoM excursion standpoint. However, the influence of this change on the proposed control variables has not been yet tested. Please note that the correlation coefficients of Table 2 were computed using the tactel configuration named *A* in Figure 17. If the coefficients are recalculated using the new arrangement, the values are, for $SUM_{CoM}$ and $F_{y}$: r=0.91[**PA1**], 0.92[**PA2**], 0.91[**PA3**], 0.88[**PA4**], 0.74[**PA5**], 0.86[**PA6**], 0.85[**PA7**], 0.81[**PA8**], 0.59[**PA9**] and 0.81[**PA10**]. For the couple formed by $SUB_{CoM}$ and $T_{z}$ they are: r=0.95[**PA1**], 0.94[**PA2**], 0.91[**PA3**], 0.94[**PA4**], 0.79[**PA5**], 0.89[**PA6**], 0.82[**PA7**], 0.40[**PA8**], 0.83[**PA9**] and 0.90[**PA10**]. As can be observed by comparing both results, the correlation is even stronger for most participants when the new tactel arrangement is used. This way, larger excursions lead to a better correspondence between the control inputs $SUM_{CoM}$ and $SUB_{CoM}$ and the magnitudes involved in the wheelchair driving through the handlebar. Besides, this tactel configuration provides the largest CoM excursion in all the handlebar height range allowed by the commercial wheelchair F35.

Yet, the fact that some correlations worsen (for example, that from **PA8** for variables $SUB_{CoM}$ and $T_{z}$) may suggest that, although there is one arrangement suitable for the majority of users, it may not be “universal”. This way, it is possible that it has to be adapted from time to time to concrete attendants.

## 7. Study of the Handlebar Grasp

As explained before, the key parameter in this system is not the pressure itself but its variation, specifically, the evolution of the CoM. This way, having an initial reference of the CoM is pivotal in order to assess how the parameter varies in subsequent measurements. The starting scenario is not that in which there is not pressure, but that in which the handlebar has just been grasped and driving related forces are not being exerted. This situation in which *there is no intention* was previously called “rest condition” and each time it is detected a process of recalibration begins. The center of mass in this situation was named $CoM_{r}$. This way, $CoM_{r}$ must be subtracted from the center of mass obtained from the captured tactile image so that it can be used to determine the user wishes:

(8) $$\begin{array}{c} CoM=CoM_{measured}-CoM_{r} \end{array}$$

where $CoM_{measured}$ is the center of mass computed for the measured tactile image. Taking into account the above equation, the control variables become:

(9) $$\begin{array}{c} SUM_{CoM}=SUM_{CoM_{measured}}-SUM_{CoM_{r}}\\ SUB_{CoM}=SUB_{CoM_{measured}}-SUB_{CoM_{r}} \end{array}$$

Hence, $SUM_{CoM}$ and $SUB_{CoM}$ are zero as long as their value is the same as that computed in rest condition.

The correct assessment of the value of $CoM_{r}$ is hence essential. When the handlebar is just grasped, during the time in which the grip is not still steady, the CoM takes transient values. A waiting time has to be introduced in order to ensure that this momentary regime is over and rest condition state has been reached. $CoM_{L_{r}}$ and $CoM_{R_{r}}$ must be stored after this waiting period. A mistake in the choice of the waiting time can have two consequences. On the one side, if it is too short, the stored $CoM_{r}$ may correspond to transient values of the CoM, what leads to a system malfunction and an erratic and unmanageable driving. On the other side, if the time is too long, the CoM stabilization process can be perceived by the attendant as contrived and uncomfortable.

An experiment was conducted (**ED**) to study the grasping process and determine a suitable waiting time once a grip is detected. In addition, it may contribute to find out if there exists some kind of pattern in the transient regime of the CoM that goes from the moment when the grip is detected until its stabilization.

### 7.1. Methods

**Figure 18:**
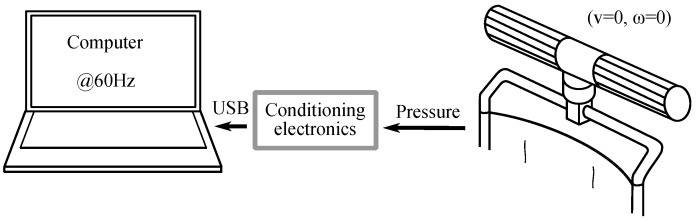
Scheme of experimental setup of the experiment **ED**.

Forty-two participants from 20 to 64 and a mean age of 34.3 years participated in the experiment **ED** after agreement and informed consent. They ignored the purpose of the study. The experimental setup explained in Section 3 was used. The height of the handlebar was 108.5 cm. As already seen, the handlebar electronics scanned the tactile arrays and sent the data to the computer at a rate of 60 Hz (see Figure 18). The PW was mechanically braked so that it was immobile. The participants were asked to stand behind the chair at such a distance that they considered natural and comfortable. They were said to grasp the handlebar after received a spoken command. They kept it grasped until they heard another command, then they released it. They performed this twice. Regarding the grip, they did it without exerting any special force beyond what was necessary to keep the handles grasped steadily.

84 trials were realized. In the data analysis, the threshold of GF above which the grip was detected was 0.03 N (t=0). Three seconds after this force threshold was reached, the grip was considered stable. It was a time long enough to have a stable CoM, as was observed experimentally.

### 7.2. Results and Discussion

#### 7.2.1. Grip Stabilization

During the time interval that goes from t=3 s to the moment before the participants received the signal to release the handlebar, the variability of the centers of mass including the data from all the tests was studied. Some statistical measures are listed in the second and third columns of Table 6.

$\Delta_{CoM}$ refers to the maximum variation of the CoM during the specified time interval, i.e., $CoM_{MAX}-CoM_{MIN}$. The first row contains the value of $\Delta_{CoM_{t>3s}}$ from the test for which it was maximum, the second is the value from the test for which it was minimum, and the third and fourth are the mean and standard deviation of this parameter when it is computed for the 84 trials. One possible way to estimate how much time is needed to reach the stable state could be to calculate how long it takes for the CoM variation to be within a specified range. Please note that the length of this range is a design choice. If it is too wide it can lead to too short waiting times and if it is very narrow to too long waiting times, with the consequences already explained. The chosen range was:

(10) $$\begin{array}{c} \Delta_{CoM_{stab.}}={\bar{\Delta }}_{CoM_{t>3s}}+2s_{\Delta_{CoM_{t>3s}}} \end{array}$$

where ${\bar{\Delta }}_{CoM_{t>3s}}$ is the mean and $s_{\Delta_{CoM_{t>3s}}}$ the standard deviation of $\Delta_{CoM_{t>3s}}$, both showed in Table 6.

Given that the distribution of the variable $\Delta_{CoM_{t>3s}}$ is approximately normal (excluding few outliers), around the 95% of the tests would have a value of this parameter inside the chosen limits. Once the threshold of the CoM variation below which the grip is considered stable ($\Delta_{CoM_{stab.}}$) was selected, the time needed to reach it was computed. On average, it was $t_{L_{stab}}=0.69$ s and $t_{R_{stab}}=0.73$ s, times for $CoM_{L}$ and $CoM_{R}$ respectively (Other values of $\Delta_{CoM_{stab.}}$ were tested, but the result was similar in terms of driving experience whereas times of CoM stabilization were longer. For example, for $\Delta_{CoM_{stab.}}={\bar{\Delta }}_{CoM_{t>3s}}$, $t_{L_{stab}}$ and $t_{R_{stab}}$ were 1.94 and 2.04 s. Adding one $s_{\Delta_{CoM_{t>3s}}}$ to the latter, the times were 1.14 and 1.24 s).

From the previous results, the initial waiting time when the handlebar grip is detected is set at 0.7 s. Once this time has passed, the centers of mass are supposed to have reached a steady value, so $CoM_{L_{r}}$ and $CoM_{R_{r}}$ are saved. Then, an audible signal is emitted to inform the user that driving can start.

#### 7.2.2. Influence of Attendant Height

The study of the user physical characteristics may be interesting. If a correspondence between the physical constitution and the parameters involved in the driving control is found, it may be exploited to improve the driving control. The data obtained in the experiment **ED** could be used to explore if the participant height has any impact on the parameters mentioned in the previous sections. For the 42 subjects, this physical feature went from 1.58 m to 1.95 m, with a mean of 1.76 m.

**Figure 19:**
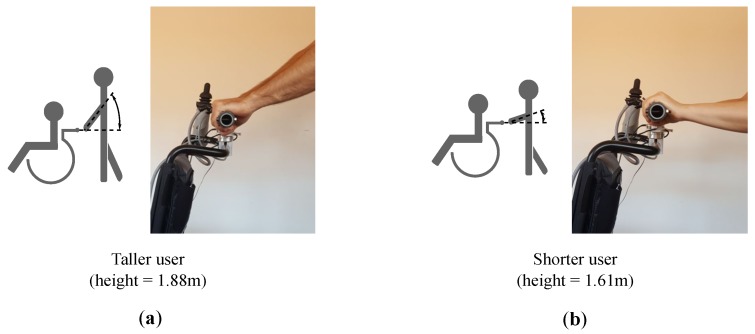
Variation of angle formed by the user arms and the handlebar with user height for a taller (**a**) and a shorter person (**b**).

**Figure 20:**
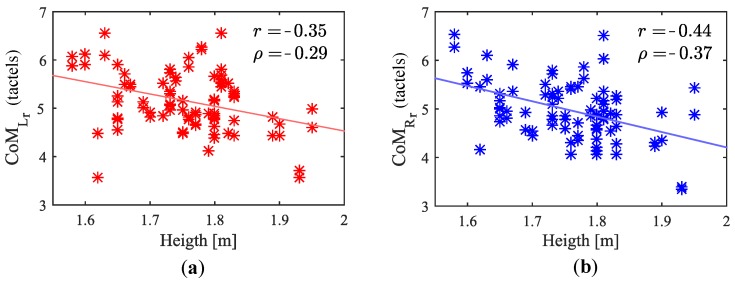
Link between (**a**) the left and (**b**) the right center of mass with stabilized grip and the attendant height. Corresponding 1st order functions superimposed.

For most participants, the angle formed by the arms when grasping the handlebar varies with height, in the way illustrated in Figure 19. This is also in line with that observed by the authors of [20]. Thus, this trend may affect the centers of mass in rest condition. Some statistical measures of this parameter are shown in Table 6, columns forth and fifth. Besides, Figure 20 plots $CoM_{L_{r}}$ and $CoM_{R_{r}}$ versus the participant height. Pearson correlation and Spearman’s rank order correlation coefficients were computed. They were r=−0.35(p=0.0011) and ρ=−0.29(p=0.0087) for the left handle and r=−0.44(p<0.001) and ρ=−0.37(p<0.001) for the right handle. The results show coefficients slightly low but not negligible (see Table 1). Graphically assessed, it seems that $CoM_{r}$ have certain tendency to increase or decrease as height is lower or higher, respectively.

**Figure 21:**
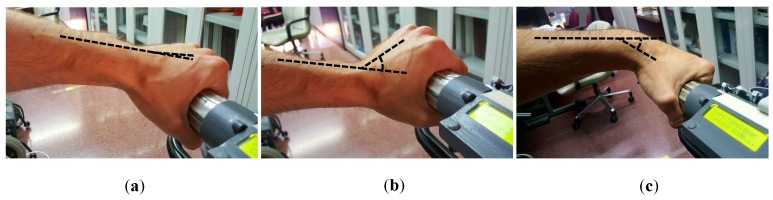
(**a**) Handle grip with an angle between the forearm and the closed hand that is almost zero. (**b**,**c**) Handle grips for which the angle is significant in both directions.

One might well wonder why the correlations are not stronger. A possible answer may lie in the angle formed by the forearm and the closed hand through the wrist joint. This angle is almost zero for many users. However, it is significant for other people in the way shown in Figure 21. When this angle is not negligible, it has an effect on $CoM_{r}$ which is similar to that caused by the variation of the assistant height. In this way, tall attendants with handle grips as that of Figure 21b will have values of $CoM_{r}$ similar to those from a shorter person whose handle grip has a zero angle. The opposite happens with short attendants for which the handle grip is like the example of Figure 21c. Their $CoM_{r}$ will be close to those from taller people with a grip with zero angle. This phenomenon may be one of the causes behind the outliers in Figure 20, which have a direct impact on the correlation values. This angle in Figure 21 seems to be an innate characteristic of the user. There were, however, cases in which the angle was affected by past injuries suffered by the participants. It was interesting the case of a subject that formed a different angle between the forearm and the hand for the left and for the right arm. According to his testimony, he had suffered a left wrist fracture some years ago.

Another observed fact is that the grip of the same attendant may be not as regular as expected. Between the first and the second test repetition, the participants were asked to walk away from the PW and to get close to it again. The aim of this instruction was to “reset” their posture behind the PW and thus to analyze if there was any change in the grip in terms of stabilization. As can be seen in Table 6, columns sixth and seventh, the mean difference of the variable $CoM_{r}$ for two grips of the same person is around one quarter of tactel. However, the results present a large dispersion. Whereas there are participants for which the $CoM_{r}$ stabilized almost in the same point in both grasps, the difference exceeds a distance of one tactel for others. Since the handlebar and user heights are fixed factors, what could be happening in cases in which this difference is large is that the same participant may be grasping with different fist-forearm angles. It may be, in turn, related to a change in the distance between the person and the chair.

**Figure 22:**
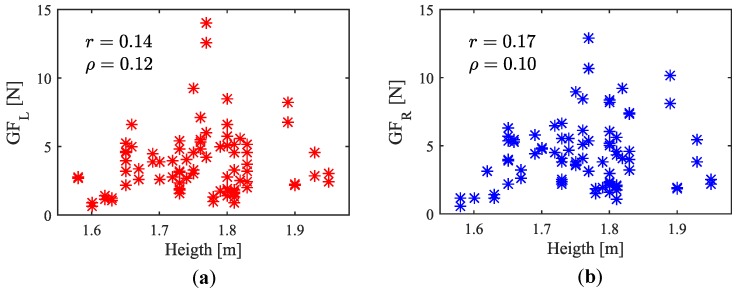
Gripping force on (**a**) the left and (**b**) the right handle versus height from participants of the experiment **ED**.

The latest results are shown in columns eighth and ninth of Table 6. They refer to the gripping force with which the participants grasped the handles in the experiment. The sample presents a large range, with minimum values near 0.5 N, a mean around 4 N and a peak of 14 N, that is far from the mean because of the sample positive skewness. Some works have shown that the hand size is directly related to the person height [26]. Others even propose the hand size as a parameter based on which the height could be estimated [27,28]. It may be hypothesized that the GF may be higher for bigger hands; they cover a larger tactile area than those that are smaller. Since the taller the people, the bigger their hands are, it would imply the existence of a link between the height and the GF. To assess this possible coupling, both parameters from the participants of the experiment **ED** are plotted together in Figure 22. Besides, Pearson correlation and Spearman’s rank order correlation coefficients were computed. The values were: r=0.14(p>0.05) and ρ=0.12(p>0.05) for the left handle and r=0.17(p>0.05) and ρ=−0.10(p>0.05) for right handle. As seen, both types of correlation are negligible. The latter numbers suggest that there is no relationship between the GF in rest condition and the attendant height.

#### 7.2.3. CoM Evolution during the Grasp Onset

In the previous sections, the focus has been on the process of stabilization of the CoM in terms of time required to have a steady value. However, the study of the CoM temporary state that starts in the very moment in which hands make contact with the handles may also provide useful information.

**Figure 23:**
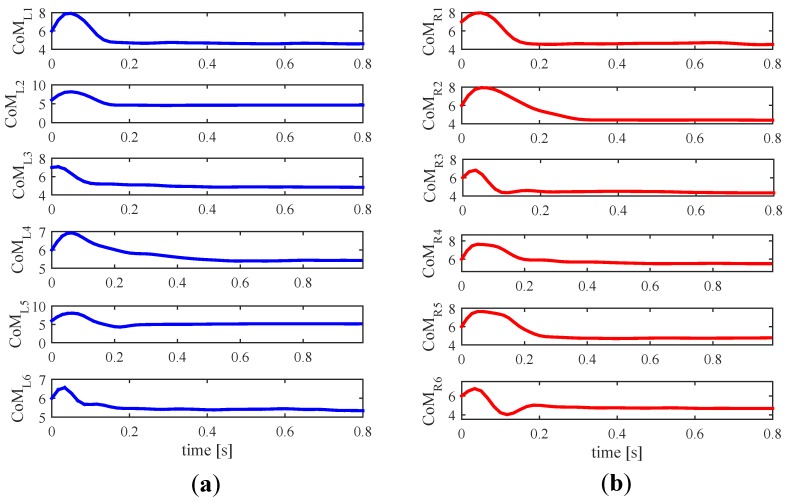
Six examples, extracted from the data of the experiment **ED**, of the evolution of (**a**) $CoM_{L}$ and (**b**) $CoM_{R}$ when the handlebar is just grasped. The parameters are expressed in tactel coordinates.

Figure 23 plots some examples of the CoM variation from the moment in which the grasp starts taking place. As the hand palm surrounds the handle, there is a certain period of time in which some tactels are pressed and others not yet; besides the output of the pressed tactels is varying. All this causes displacements of the CoM. The curves depicted in Figure 23 are similar to those found in approximately 80% of the tests of the experiment **ED**. This way, it may reasonable to think that there is an underlying pattern or tendency.

**Figure 24:**
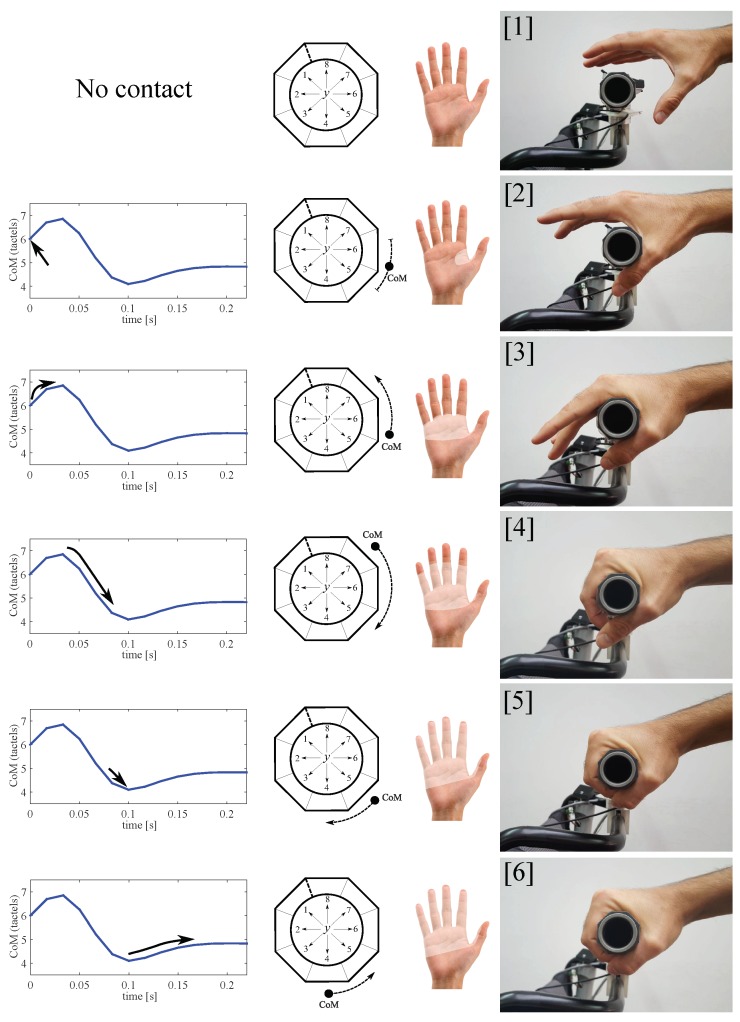
Process of the handlebar grasp (ordered from top to bottom). The lighter area in the open hand represents the contact with the handle in each step ([1]–[6]). Please note that the tactel arrangement **E** was used.

To explain how the grasp takes place, it is interesting to match the parts of the curves with the pressure exerted by the different areas of the hand. Figure 24 is helpful in this regard. In this figure, the grasping process has been represented, starting from a situation in which there is no contact between the tactels and the hand ([1]) and ending with a grasp in which the CoM variation starts decreasing([6]). The hand makes contact with the handle in ([2]) and it surrounds it totally in ([5]). The case illustrated in Figure 23 is that of some tests that present a little rebound just when the gripping force is maximum ([5] and [6]) (for example, $CoM_{R3}$ or $CoM_{R6}$). For others, the CoM starts stabilizing after step ([3]) (for example, $CoM_{L2}$ or $CoM_{R2}$).

As said, the pattern (with its subtle variations) has been seen in 80% of the tests of the experiment **ED**. This way, its identification may be useful to distinguish if a just made contact comes from a hand grip or it has been caused by an object or a person with an intention distinct from grasping, for example, just leaning his or her forearms on the handlebar to rest. On another note, according to the results of the experiment **ED** the process of the handlebar release is basically symmetric with respect to that observed when the handlebar was grasped (see Figure 23). This means that the release takes place in reverse order to that showed in Figure 24, i.e., following the steps from [6] to [1].

## 8. Conclusions

This paper has covered a set of key aspects regarding the use of tactile sensors to extract control inputs in a handlebar shape interface. An experiment has been conducted to assess the capacity to identify and quantify the user intention of two variables based on the center of mass computed for the left and right tactile handle. They are the sum and subtraction of the latter parameters. The results have validated this assumption showing a strong correlation between the first of the variables and the force exerted when pulling and pushing the handlebar. A high correlation has also been found between the center of mass subtraction and the torque involved in turn maneuvering. However, the performance of both variables as control inputs is influenced by some human factors. The first of them is the gripping force. The analysis of the results of the previous experiment has proven that the proposed input variables worsen as user intention predictors as the average force on the handlebar increases. A second experiment has been carried out to study the effect of this force on the excursion of the center of mass. In this case, the results have shown that the higher the gripping force, the lower the variation range of the center of mass is. The data of the experiment have been used to build gripping force dependent curves, that act as variable gain functions to minimize this unwanted effect on the control inputs. Moreover, the spatial configuration of the tactels inside the tactile sensors has been analyzed. The optimal tactel arrangement has been identified by performing a third experiment where typical maneuvers were carried out. Finally, the grasping process has been explored. A transient phase that starts just when the hands make contact has been studied. The duration of this phase has been determined through a fourth experiment. It helps set the instant from which the center of mass is properly stabilized and is available for the computation of the control inputs. The results of the same experiment have been used to study whether the user height influences the center of mass location. Although the data seem to confirm a moderate influence, the angle between the forearm and the hand introduce has also to be taken into account. Lastly, the analysis of the data has provided the identification of a pattern in the evolution of the center of mass when the handlebar is grasped and released. Its recognition is useful to distinguish between human grasp and other kind of contacts.

## Figures and Tables

**Table 1:** Rule of thumb for correlation interpretation.

Size of Correlation	Interpretation
0.9 to 1/−0.9 to −1	Very high positive/negative correlation
0.7 to 0.9/−0.7 to −0.9	High positive/negative correlation
0.5 to 0.7/−0.5 to −0.7	Moderate positive/negative correlation
0.3 to 0.5/−0.3 to −0.5	Low positive/negative correlation
0 to 0.3/0 to −0.3	Negligible

**Table 2:** Results for the tests of the experiment **EA**: Pearson correlation coefficients for <$SUM_{CoM}$,$F_{y}$> and <$SUB_{CoM}$,$T_{z}$> (2nd and 4th columns) and their respective 1st order approximations (3rd and 5th columns).

Participant	$r_{<{\mathrm{SUM}}_{\mathrm{CoM}},F_{y}>}$	$F_{y}={\mathrm{aSUM}}_{\mathrm{CoM}}+b$	$r_{<{\mathrm{SUB}}_{\mathrm{CoM}},T_{z}>}$	$T_{z}={\mathrm{cSUB}}_{\mathrm{CoM}}+d$
PA1	0.91 (p<0.001)	$F_{y}=0.70SUM_{CoM}-87.69$	0.95 (p<0.001)	$T_{z}=0.17SUB_{CoM}+1.40$
PA2	0.83 (p<0.001)	$F_{y}=0.98SUM_{CoM}-113.06$	0.84 (p<0.001)	$T_{z}=0.31SUB_{CoM}+1.21$
PA3	0.77 (p<0.001)	$F_{y}=1.93SUM_{CoM}-266.37$	0.85 (p<0.001)	$T_{z}=0.60SUB_{CoM}+2.32$
PA4	0.89 (p<0.001)	$F_{y}=1.52SUM_{CoM}-218.59$	0.88 (p<0.001)	$T_{z}=0.39SUB_{CoM}+1.79$
PA5	0.71 (p<0.001)	$F_{y}=1.38SUM_{CoM}-198.80$	0.70 (p<0.001)	$T_{z}=0.50SUB_{CoM}+4.18$
PA6	0.83 (p<0.001)	$F_{y}=1.61SUM_{CoM}-206.18$	0.80 (p<0.001)	$T_{z}=0.55SUB_{CoM}-1.10$
PA7	0.56 (p<0.001)	$F_{y}=4.26SUM_{CoM}-653.25$	0.68 (p<0.001)	$T_{z}=1.10SUB_{CoM}-0.50$
PA8	0.80 (p<0.001)	$F_{y}=3.96SUM_{CoM}-588.03$	0.67 (p<0.001)	$T_{z}=1.05SUB_{CoM}+5.25$
PA9	0.78 (p<0.001)	$F_{y}=2.11SUM_{CoM}-299.01$	0.84 (p<0.001)	$T_{z}=0.61SUB_{CoM}+1.48$
PA10	0.85 (p<0.001)	$F_{y}=1.79SUM_{CoM}-223.17$	0.82 (p<0.001)	$T_{z}=0.44SUB_{CoM}+1.40$

**Table 3:** Gradients of the mean of the linear approximations of the tests inside groups associated with pushing/pulling maneuvers (second column for $CoM_{L}$ and third for $CoM_{R}$). Gradient of $SUM_{CoM}$ computed from the latter (fourth column). Gradients of the mean of the linear approximation of those tests inside groups linked to turns (sixth column for $CoM_{L}$ and seventh for $CoM_{R}$). Gradient of $SUB_{CoM}$ computed using the latter (eight column).

Group	${\bar{a}}_{L_{\mathrm{PP}}}$	${\bar{a}}_{R_{\mathrm{PP}}}$	$m_{{\mathrm{SUM}}_{\mathrm{CoM}}}\left({\bar{a}}_{L_{\mathrm{PP}}}+{\bar{a}}_{R_{\mathrm{PP}}}\right)$	Group	${\bar{a}}_{L_{T}}$	${\bar{a}}_{R_{T}}$	$m_{{\mathrm{SUB}}_{\mathrm{CoM}}}\left({\bar{a}}_{L_{T}}-{\bar{a}}_{R_{T}}\right)$
$G_{PP_{1}}$	−0.1323	−0.1459	−0.2782	$G_{T_{1}}$	1.0470	−1.0525	2.0995
$G_{PP_{2}}$	−0.067	−0.1201	−0.1871	$G_{T_{2}}$	0.4729	−0.6288	1.1017
$G_{PP_{3}}$	−0.0547	−0.06	−0.1147	$G_{T_{3}}$	0.3053	−0.1765	0.4818
$G_{PP_{4}}$	−0.0405	−0.039	−0.0795	$G_{T_{4}}$	0.1429	−0.1547	0.2975
$G_{PP_{5}}$	−0.0295	−0.0196	−0.0491	$G_{T_{5}}$	0.0320	−0.0390	0.0711
$G_{PP_{6}}$	−0.0111	−0.0098	−0.0209	$G_{T_{6}}$	0.0296	−0.0293	0.0589

**Table 4:** Number of tests for which the use of each tactel arrangement led to the maximum CoM excursion with a handlebar height of $h_{1}=98.5$ cm.

**All Maneuvers (**N=48**)**	${\mathrm{CoM}}_{A}$	${\mathrm{CoM}}_{B}$	${\mathrm{CoM}}_{C}$	${\mathrm{CoM}}_{D}$	${\mathrm{CoM}}_{E}$	${\mathrm{CoM}}_{F}$	${\mathrm{CoM}}_{G}$	${\mathrm{CoM}}_{H}$
Left handle largest exc.	3	1	0	2	22	20	0	0
Right handle largest exc.	0	1	2	5	30	10	0	0
**Pushing/Pulling (**N=24**)**	${\mathrm{CoM}}_{A}$	${\mathrm{CoM}}_{B}$	${\mathrm{CoM}}_{C}$	${\mathrm{CoM}}_{D}$	${\mathrm{CoM}}_{E}$	${\mathrm{CoM}}_{F}$	${\mathrm{CoM}}_{G}$	${\mathrm{CoM}}_{H}$
Left handle largest exc.	0	1	0	0	15	8	0	0
Right handle largest exc.	0	1	2	2	17	2	0	0
**Turns (**N=24**)**	${\mathrm{CoM}}_{A}$	${\mathrm{CoM}}_{B}$	${\mathrm{CoM}}_{C}$	${\mathrm{CoM}}_{D}$	${\mathrm{CoM}}_{E}$	${\mathrm{CoM}}_{F}$	${\mathrm{CoM}}_{G}$	${\mathrm{CoM}}_{H}$
Left handle largest exc.	3	0	0	2	7	12	0	0
Right handle largest exc.	0	0	0	3	13	8	0	0

($h_{1}=98.5$ cm).

**Table 5:** Number of tests for which the use of each tactel arrangement led to the maximum CoM excursion with a handlebar height of $h_{2}=108$ cm.

**All Maneuvers (**N=48**)**	${\mathrm{CoM}}_{A}$	${\mathrm{CoM}}_{B}$	${\mathrm{CoM}}_{C}$	${\mathrm{CoM}}_{D}$	${\mathrm{CoM}}_{E}$	${\mathrm{CoM}}_{F}$	${\mathrm{CoM}}_{G}$	${\mathrm{CoM}}_{H}$
Left handle largest exc.	1	0	1	1	28	17	0	0
Right handle largest exc.	0	2	2	2	26	16	0	0
**Pushing/Pulling (**N=24 **)**	${\mathrm{CoM}}_{A}$	${\mathrm{CoM}}_{B}$	${\mathrm{CoM}}_{C}$	${\mathrm{CoM}}_{D}$	${\mathrm{CoM}}_{E}$	${\mathrm{CoM}}_{F}$	${\mathrm{CoM}}_{G}$	${\mathrm{CoM}}_{H}$
Left handle largest exc.	0	0	1	1	15	7	0	0
Right handle largest exc.	0	2	2	0	13	7	0	0
**Turns (**N=24**)**	${\mathrm{CoM}}_{A}$	${\mathrm{CoM}}_{B}$	${\mathrm{CoM}}_{C}$	${\mathrm{CoM}}_{D}$	${\mathrm{CoM}}_{E}$	${\mathrm{CoM}}_{F}$	${\mathrm{CoM}}_{G}$	${\mathrm{CoM}}_{H}$
Left handle largest exc.	1	0	0	0	13	10	0	0
Right handle largest exc.	0	0	0	2	13	9	0	0

($h_{2}=108$ cm).

**Table 6:** Maximum, minimum, mean and standard deviation for: maximum variation of the CoM from 3 s after the grasp is detected until just before the user releases the handles (2nd and 3rd columns), the CoM in rest condition (4th and 5th columns), difference between the value $CoM_{r}$ in two consecutive grips of the same user (6th and 7th columns) and gripping force in rest condition (8th and 9th columns).

Stat. Meas.	$\Delta_{{\mathrm{CoM}}_{t>3s}}$		${\mathrm{CoM}}_{r}$		${\mathrm{CoM}}_{r_{k}}-{\mathrm{CoM}}_{r_{k+1}}$		GF	
**(**N=84**)**	L. Handle	R. Handle	L. Handle	R. Handle	L. Handle	R. Handle	L. Handle	R. Handle
**Max.**	0.37	0.33	6.55	6.54	1.04	1.31	14.06	12.87
**Min.**	0.02	0.02	3.56	3.35	0.01	0.005	0.62	0.59
**Mean**	0.11	0.09	5.15	4.97	0.26	0.26	3.85	4.28
**Std. Dev.**	0.07	0.06	0.62	0.61	0.27	0.26	2.45	2.45

(Parameters expressed in Number of Tactels, except GF that is given in Newton).

## Abbreviations

The following abbreviations are used in this manuscript:

Tactel	Tactile element
PW	Powered wheelchair
CoM	Center of mass
$CoM_{L}$	Center of mass computed for the left handle
$CoM_{R}$	Center of mass computed for the right handle
GF	Gripping force
$SUB_{CoM}$	Subtraction of $CoM_{L}$ and $CoM_{R}$
$SUM_{CoM}$	Sum of $CoM_{L}$ and $CoM_{R}$
$CoM_{r}$	Center of mass in rest condition. Reference value to assess the CoM deviations
$F_{y}$	Force exerted on the handlebar to carry out push and pull maneuvers
$T_{z}$	Torque exerted on the handlebar when carrying out turns
